# Multi-Elemental Analysis of Edible Insects, Scorpions, and Tarantulas from French (Online) Market and Human Health Risk Assessment Due to Their Consumption: A Pilot Study

**DOI:** 10.3390/foods13152353

**Published:** 2024-07-26

**Authors:** Yulianna Holowaty, Axelle Leufroy, Clément Mazurais, Diane Beauchemin, Petru Jitaru

**Affiliations:** 1 Department of Chemistry, Queen’s University, 90 Bader Lane, Kingston, ON K7L 3N6, Canada; 18yzh@queensu.ca (Y.H.); diane.beauchemin@queensu.ca (D.B.); 2 Laboratory for Food Safety, University Paris East Creteil, Anses, F-94700 Maisons-Alfort, France; axelle.leufroy@anses.fr (A.L.); clement.mazurais@anses.fr (C.M.)

**Keywords:** trace elements, ICP-MS, edible insects, France, risk assessment

## Abstract

Edible insects are becoming increasingly popular as protein alternatives to traditional animal-based products. As such, information on their elemental composition is important to ensure they are safe for human consumption. This article describes the development and validation of a rapid, reliable method for the simultaneous determination of 19 elements (Al, As, B, Ba, Ca, Cd, Co, Cr, Cu, Fe, K, Mg, Mn, Mo, Na, Pb, Se, Sr, and Zn) in edible insects by inductively coupled plasma mass spectrometry (ICP-MS) following closed vessel microwave digestion. The method was validated using three insect certified reference materials, namely black soldier fly larvae meal (BFLY-1), cricket flour (KRIK-1), and mealworm powder (VORM-1). The method was applied to analyze twelve different (whole) insect species. The maximum amount of each sample was calculated for As, Cd, and Pb with respect to their provisional tolerable daily intake values established by the Food and Agricultural Organization/World Health Organization. Most of the samples, except for scorpions and tarantulas, were safe to consume at large doses (1000–10,000 insects per day). Furthermore, most of the samples contained high levels of Fe, K, Na, and Zn, providing a preliminary overview of the nutritional profile of these novel protein alternatives.

## 1. Introduction

Entomophagy is a term used to describe the practice of eating insects [1]. While common in some countries, Western regions are still overcoming psychological and cultural barriers [2]. Recent efforts have been made to eliminate these barriers and promote the consumption of insects as an alternative protein source, given that the world’s population is estimated to reach approximately 9.7 billion by 2050 and a shortage of current animal-based proteins is expected [3,4,5]. An increasing number of edible insect products are emerging on the market, providing consumers with environmentally friendly and nutritious options, taking into account that insects consume less water and land, emit less greenhouse gases compared to livestock, and they contain proteins, essential vitamins, and minerals [6,7,8,9,10]. However, information on the total elemental composition of these commercially available edible insects is required to ensure their safety for human consumption.

Trace elements in the human body, which are generally present at levels < 250 μg g^−1^ are classified as essential, nonessential, and potentially toxic, depending on the dose and duration of intake [11,12,13]. Essential trace elements play a vital role in human health and functions. They include Cu and Fe, which participate in energy metabolism via oxidation-reduction reactions, and Fe enables transport of oxygen throughout the body via the formation of hemoglobin [11,13]. Meanwhile, potentially toxic elements (PTEs) include As, Cd, Hg, and Pb, which can lead to cancer and damage to the nervous system and organs when consumed at high doses for long periods of time [11,13,14,15,16]. There is a possibility that insects, such as the black soldier fly larvae (BSFL, *Hermetia illucens*) that convert organic matter into biomass, are bioaccumulating these PTEs from their feed, posing health risks for humans if ingested [17,18]. To minimize these risks, trace element levels in edible insects must be monitored. 

As the emergence of insect-based proteins is relatively new, there is little information on their elemental composition in the current literature. Although techniques such as particle-induced X-ray emission (micro PIXE), instrumental neutron activation analysis (INAA), and wavelength dispersive X-ray fluorescence (WDXRF) spectrometry [19,20,21,22,23,24] have been previously used for the determination of elements in various insect samples, inductively coupled plasma optical emission spectrometry (ICP-OES) and inductively coupled plasma mass spectrometry (ICP-MS) allow for simultaneous multi-elemental analysis [17,18,25,26,27,28,29]. Notably, ICP-MS provides lower detection limits and greater sensitivity, making it the suitable choice for this type of research [30,31].

The aim of this study was to develop and validate a rapid and reliable method for the determination of minor, major, potentially toxic, and essential elements in a selection of commercially available edible insects using ICP-MS. For this purpose, nineteen trace elements were studied: Al, As, B, Ba, Ca, Cd, Co, Cr, Cu, Fe, K, Mg, Mn, Mo, Na, Pb, Se, Sr, and Zn. As the proposed method has not previously been applied to such matrices, it was validated through the analysis of several insect certified reference materials (CRMs) before being applied to the analysis of real-life insect samples. 

## 2. Materials and Methods

### 2.1. Instrumentation

The samples were digested using a closed microwave system (Mutiwave 7000, Anton Paar, Courtaboeuf, France).

An Agilent 8900 ICP tandem mass spectrometry (ICP-MS/MS) instrument (Agilent Technologies, Courtaboeuf, France) equipped with a MicroMist concentric nebulizer and Scott-type double-pass quartz spray chamber was used for analysis. Sample digests were introduced directly into the instrument via a peristaltic pump from tubes connected to an SPS 4 autosampler (Agilent Technologies, Courtaboeuf, France). The operating conditions are detailed in Table 1. These parameters were optimized daily by performing short-term stability tests with the tuning solution (1 mg L^−1^) in both detection modes (standard and collision mode) to ensure maximum sensitivity and minimal interference from oxide (CeO^+^/Ce^+^ < 1.2%) and doubly charged ions (Ce^2+^/Ce^+^ < 2%). The signals were obtained using Scientific MassHunter software (Agilent Technologies) and the raw data were processed using Excel.

### 2.2. Chemicals and Reagents

Ultrapure water (18.2 MΩ cm, Millipore Milli-Q™, Merck Millipore, Saint Quentin en Yvelines, France) and HNO_3_ (67% *v*/*v*, Suprapur, VWR, Fontenay sous-Bois, France) were used throughout the study. High purity argon (99.996%, Linde Gas, Montereau-Fault-Yonne, France) was used for the plasma, auxiliary, and nebulizer gases.

Stock solutions containing 1000 mg L^−1^ of each analyte (Al, As, B, Ba, Ca, Cd, Co, Cr, Cu, Fe, K, Mg, Mn, Mo, Na, Pb, Se, Sr, and Zn) were purchased from LGC Standards (Molsheim, France) and were used to prepare calibration standard solutions daily in 6% (*v*/*v*) HNO_3_.

Internal standard solutions were prepared using 1000 mg L^−1^ standard stock solutions of Y, Re, and Bi that were purchased from LGC Standards. These solutions were added to all samples, calibration standards, and blanks to compensate for drift. 

A tuning solution and a Factor P/A solution were prepared separately using multi-element solutions (Agilent Technologies) to ensure optimal instrument sensitivity over a wide range of masses and linear response of the detector between pulse and analog detection modes, respectively. 

Solutions of HNO_3_ at 6% (*v*/*v*) and 10% (*v*/*v*) were used for rinsing the ICP-MS/MS system between analyses.

### 2.3. Reference Materials and Samples

Three insect-based CRMs provided by the National Research Council Canada (Ottawa, ON, Canada) were used throughout the study to validate the method, namely: BFLY-1 (black soldier fly larvae meal), KRIK-1 (cricket flour), and VORM-1 (mealworm powder). The mussel tissue ERM-CE278k CRM from the Institute for Reference Materials and Measurements (LGC Standards) was also used throughout the study as an internal quality control sample.

Fourteen dry samples including authorized edible crickets (*Acheta domesticus*), worms (*Tenebrio molitor*), ants (*Atta laevigata*), water bugs (*Nepidae*), locusts (*Locusta migratoria*), scorpions (*Heterometrus longimanus*), and tarantulas (*Haplopeima albostriatum*) were purchased from the French market and online. To ensure sufficient quantities for analysis, multiple packages of some samples were purchased. Although these packages originated from the same batch, they were each treated as a single sample to distinguish any variations between packages, which led to the analysis of 27 samples (see Table 2 for details). Information on the life stage and origin of the samples is not available.

### 2.4. Analytical Procedures

#### 2.4.1. Sample Preparation

Each sample was removed from its original packaging and stored at room temperature in a closed polyethylene tube while awaiting preparation for analysis. As most packages contained multiple insects, the total weight (in g) was measured. From this, the weight per insect (in g) was approximated by weighing 20 whole insects.

Each sample was then homogenized using a BM500 benchtop laboratory ball mill (Anton Paar, Courtabœuf, France) and three 10 mm agate beads. The grinding frequency and time varied depending on the type of insect. Those with hard exoskeletons (scorpions, water bugs, and tarantulas) were ground at 30 Hz for 2 min, whereas others were ground at 25 Hz for 45 s.

#### 2.4.2. Multi-Elemental Analysis by ICP-MS/MS

Approximately 0.3 g of homogenous sample was weighed in a quartz digestion vial, to which 3 mL of HNO_3_ (67%, *v*/*v*) and 3 mL of ultrapure water were added. The samples were then completely digested using a closed microwave digestion system for 30 min at 250 °C and a pressure of 140 bar. Once cooled to room temperature, the sample solutions were transferred into 50 mL polyethylene flasks and 100 µL of a mixture of internal standard solution (Y, Re, and Bi) were added before final dilution to 50 mL with ultrapure water and subsequent analysis by ICP-MS.

#### 2.4.3. Data Processing

Concentrations were expressed in mg kg^−1^. Calibration curves were produced for each analysis series to verify linearity (R^2^ ≥ 0.995). Humidity was measured in all three insect CRMs to correct the result. A Grubbs test at the 95% confidence level was performed to identify and remove any outliers in the experimentally obtained data.

To determine the method’s accuracy when analyzing the three insect CRMs, the difference between the certified and measured values (Δ*_m_*) were compared to their combined uncertainties (*U*_Δ_) using the Equations (1)–(3) [32,33]:

(1) $${∆}_{m}=\left|c_{m}-c_{CRM}\right|$$

(2) $$u_{∆}=\sqrt{s_{m}^{2}+s_{CRM}^{2}}$$

(3) $$U_{∆}=k\cdot u_{∆}$$

where *c_m_* is average measured concentration, *c_CRM_* is certified concentration, *s_m_* is standard deviation of the measured value, and *s_CRM_* is uncertainty of the certified value. Note, a coverage factor (*k*) of 2 was used, corresponding to 95% confidence level.

## 3. Results and Discussion

### 3.1. Method Validation

**Figure 1:**
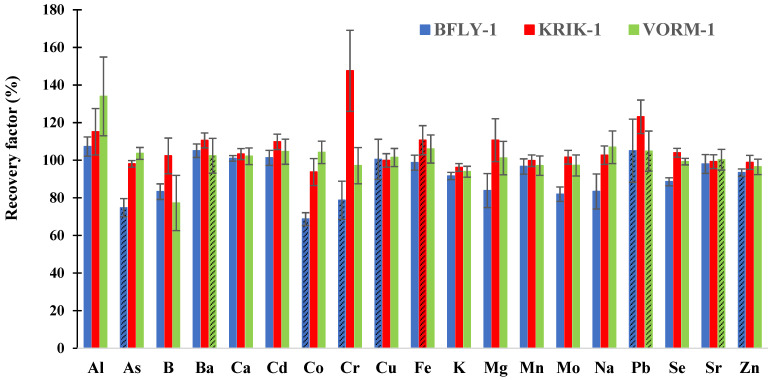
Average recovery factors (%) and the relative standard deviation (*n* = 8, patterned bars or *n* = 9) obtained for the analysis of BFLY-1, KRIK-1, and VORM-1 CRMs by ICP-MS.

To ensure that the proposed method is applicable to insect matrices, three CRMs (BFLY-1, KRIK-1, and VORM-1) were analyzed in triplicates on three different days (*n* = 9, unless otherwise specified) over a period of seven weeks. The comparison of the average total concentration and standard deviation determined for each of the 19 elements in the three CRMs and the corresponding certified value is provided in Figure 1.

Overall, most of the elements had average percent recoveries ranging from 90 to 110% for all three CRMs, hence confirming the method’s fit for the analysis of insects. Furthermore, upon comparing Δ*_m_* with *U*_Δ_ (Table S1), all the elements except for Co, K, and Se in BFLY-1, were found to have Δ*_m_* values lesser than *U*_Δ_, indicating that there were no statistically significant differences between the measured and certified concentrations.

### 3.2. Analysis of Commercially Available Edible Insects

The levels of 19 elements measured in commercially available edible insects are presented as the following: PTEs, major elements, essential elements, and others (see Table 3, Table 4, Table 5 and Table 6, respectively). It is worth to note that amongst the species analyzed in this study, only four are currently authorized as novel food in the European Union (EU), namely the mealworm (*Tenebrio molitor*), the lesser mealworm (*Alphitobius diaperionus*), the locust (*Locusta migratoria*), and the cricket (*Acheta domesticus*) [34].

The Food and Agricultural Organization/World Health Organization established provisional tolerable daily intake (PTDI) values for As, Cd, and Pb of 2.10 µg kg^−1^, 0.82 µg kg^−1^, and 3.57 µg kg^−1^ bodyweight per day, respectively, in this type of foodstuffs [35,36]. The maximum amount of whole edible insects that can be safely consumed daily for a 60 kg adult and an 18 kg child was calculated using the approximated weight per insect (in g) and is summarized in Table 3.

Based on the measured concentrations, it appears that most of the samples, notably the crickets, locusts, and worms, can be safely consumed at rather large quantities (from 1000 to 100,000 individuals) by both children and adults. However, the maximum number of scorpions that can be consumed in terms of Cd were significantly lower than the other samples, with 12–32 recommended for adults and 4–10 for children.

Moreover, in the case of tarantula samples, the consumption of only 1 individual per day is recommended for children and 2–3 for adults. However, this finding should not pose too much concern considering the context in which insects are being considered as a protein alternative. For instance, in Europe, insects are marketed as a food ingredient, to be added in crackers, pasta, etc. [34].

With regard to the samples originating from the same lot, similar contamination patterns were observed for the ants, crickets 1–3 and 7–8, and worms 4–6 samples. Contrarily, measured results varied significantly between the following samples (see Table 3, Table 4, Table 5 and Table 7): crickets 5–6, locusts 1–2, scorpions 3–4, tarantulas 1–3, water bug 1–2, and worms 1–3. Larger variances observed in scorpions, tarantulas, and water bugs can be explained by the number of individuals in each lot. In fact, especially for these species, one lot was composed of only one or two individuals. 

Table 4 summarizes the levels of major (naturally abundant) elements (Na, K, Ca, and Mg) in each sample. Overall, high levels of K were observed for all the samples (from 4- to 10-fold compared to Ca, Mg, and Na), suggesting that insects are rich in this element. This is beneficial as K plays a role in proper kidney and heart regulation, muscle contraction, and nerve transmission [37]. 

**Table 3:** Concentrations ± standard deviation (*n* = 2; µg kg^−1^) of potentially toxic elements in edible insects, scorpions, and tarantulas (with corresponding sample name) by ICP-MS as well as the number of individual of each type that can be safely consumed (per day) in relation to provisional tolerable daily intake (PTDI) ^a^ for human adults ^b^ and children ^c^.

Sample	As			Cd			Pb		
	Measured	Maximum Recommended Consumption (Number per Day)		Measured	Maximum Recommended Consumption (Number per Day)		Measured	Maximum Recommended Consumption (Number per Day)	
		Adults	Children		Adults	Children		Adults	Children
Ants									
1	43.86 ± 0.66	401	120	85.4 ± 1.7	80	24	106.8 ± 5.4	280	84
2	44.13 ± 0.77	429	129	79.60 ± 0.15	93	28	104.1 ± 1.2	309	93
Crickets									
1	7.70 ± 0.40	145,196	43,559	36.89 ± 0.39	11,834	3550	18.296 ± 0.018	103,882	31,165
2	6.97 ± 0.18	178,103	53,431	38.95 ± 0.72	12,445	3733	15.18 ± 0.51	139,021	41,706
3	6.449 ± 0.084	182,939	54,882	29.35 ± 0.92	15,696	4709	16.7 ± 2.9	120,097	36,029
4	12.42 ± 0.65	41,399	12,420	13.75 ± 0.11	14,602	4381	36.62 ± 0.81	23,870	7161
5	3.6 ± 2.7	271,389	81,417	73 ± 10	5182	1555	53.6 ± 2.4	30,729	9219
6	2.622 ± 0.086	423,019	126,906	65.90 ± 0.49	6572	1972	33.4 ± 3.8	56,454	16,936
7	19.57 ± 0.73	13,757	4127	76.07 ± 0.92	1382	415	96.9 ± 3.0	4723	1417
8	21.07 ± 0.67	12,726	3818	76.6 ± 3.8	1367	410	62.0 ± 5.6	7352	2206
Locusts									
1	35.0 ± 1.0	8137	2441	31.78 ± 0.34	3499	1050	50.194 ± 0.060	9645	2894
2	47.44 ± 0.12	4912	1474	40.9 ± 2.3	2225	667	73.4 ± 1.9	5397	1619
Scorpions									
1	725 ± 36	62	19	539 ± 22	32	10	235 ± 14	324	97
2	383.5 ± 2.4	200	60	2272 ± 19	13	4	123.6 ± 3.1	1053	316
3	233.1 ± 3.3	226	68	1001 ± 38	21	6	162 ± 17	554	166
4	192.20 ± 0.23	127	38	771.406 ± 0.046	12	4	64.6 ± 9.7	643	193
Tarantulas									
1	93.78 ± 0.51	65	19	13,200 ± 97	2	1	633.3 ± 6.3	163	49
2	191.8 ± 4.2	326	98	9650 ± 190	3	1	464.79 ± 0.89	229	69
3	120.8 ± 1.3	476	143	8582 ± 48	3	1	1064 ± 27	92	28
Water bug									
1	59.31 ± 0.64	681	204	3.46 ± 0.15	4556	1367	60.96 ± 0.98	1126	338
2	86.4 ± 2.6	416	125	6.98 ± 0.14	2009	603	104.9 ± 1.4	582	175
Worms									
1	39.73 ± 0.76	75,962	22,789	82.6 ± 2.1	14,267	4280	10.8 ± 1.7	475,050	142,515
2	470 ± 15	1483	445	53.9 ± 4.2	5049	1515	87.3 ± 5.2	13,571	4071
3	21.25 ± 0.62	20,567	6170	16.777 ± 0.061	10,172	3052	7.425 ± 0.011	100,064	30,019
4	25.65 ± 0.79	119,520	35,856	79.25 ± 0.76	15,105	4532	7.5 ± 2.5	694,891	208,467
5	26.50 ± 0.53	138,018	41,405	83.5 ± 2.9	17,104	5131	6.03 ± 0.28	1,031,129	309,339
6	25.66 ± 0.12	103,376	31,013	77.7 ± 2.8	13,331	3999	6.32 ± 0.39	713,524	214,057

^a^ PTDI levels for As, Cd, and Pb are 2.10, 0.82, and 3.57 µg kg^−1^ bw^−1^ day^−1^, respectively. ^b^ weight of 60 kg. ^c^ weight of 18 kg.

Although natural samples (free from additives or other ingredients besides the insect) were purchased for this study, some were found to have salt added to them (see ingredients listed in Table 2). This was reflected by high levels of Na being reported in the scorpion and tarantula samples (up to 10,597 and 40,454 mg kg^−1^, respectively). Sodium, being an essential nutrient, helps maintain plasma volume, acid-base balance, nerve transmissions, and healthy cell functions [38].

**Table 4:** Concentrations ± standard deviation (*n* = 2; mg kg^−1^) of major elements in edible insects, scorpions, and tarantulas (with corresponding sample name) measured by ICP-MS.

Sample	Ca	K	Mg	Na
Ants				
1	539 ± 62	681 ± 2	187 ± 2	1135 ± 5
2	458 ± 87	677 ± 5	184 ± 3	1117 ± 22
Crickets				
1	1268 ± 13	7567 ± 150	1215 ± 11	2577 ± 9
2	1399 ± 16	8308 ± 120	1316 ± 34	2835 ± 66
3	1397 ± 21	8538 ± 79	1314 ± 43	2874 ± 33
4	1798 ± 12	5263 ± 61	727 ± 1	1771 ± 12
5	1053 ± 66	9116 ± 330	746 ± 12	2424 ± 130
6	1034 ± 53	9078 ± 220	801 ± 10	2448 ± 15
7	496 ± 2	7017 ± 26	867 ± 13	1259 ± 12
8	411 ± 8	6559 ± 1	777 ± 2	1162 ± 6
Locusts				
1	559 ± 24	6510 ± 150	749 ± 34	937 ± 57
2	481 ± 21	6350 ± 460	658 ± 4	860 ± 19
Scorpions				
1	1149 ± 52	5946 ± 510	430 ± 7	10,597 ± 1009
2	1792 ± 14	5101 ± 37	542 ± 7	4152 ± 23
3	2102 ± 42	6903 ± 4	784 ± 28	6150 ± 7
4	1163 ± 4	4883 ± 53	417 ± 4	4495 ± 28
Tarantulas				
1	5372 ± 14	1298 ± 10	1944 ± 43	32,232 ± 320
2	2518 ± 74	1794 ± 38	1984 ± 12	27,079 ± 620
3	2748 ± 26	2220 ± 12	1765 ± 40	40,454 ± 260
Water bug				
1	632 ± 2	3950 ± 4	695 ± 5	3150 ± 2
2	865 ± 18	4041 ± 18	614 ± 10	5113 ± 49
Worms				
1	401 ± 10	4474 ± 250	1894 ± 69	521 ± 8
2	1132 ± 1	7462 ± 130	2565 ± 11	126 ± 1
3	788 ± 67	6040 ± 29	1343 ± 17	670 ± 6
4	499 ± 1	8335 ± 300	3074 ± 6	980 ± 10
5	492 ± 6	8533 ± 400	2896 ± 70	982 ± 38
6	501 ± 15	8294 ± 50	2959 ± 3	976 ± 10

Table 7 compares the results obtained in previous studies with those obtained in this work for potential toxic and major elements. Many results are comparable despite the samples being from different origins, prepared differently, etc. For example, two out of three samples of cricket in the previous study had up to 10–38% of seasonings, whereas only whole dehydrated crickets were analyzed in this work. Yet, except for As, the levels of Cd, Pb, Ca, Mg, K, and Na are fairly similar. The largest differences were observed for water bugs, which were from India in the previous study. 

As can be seen in Table 5, high Cu levels were measured in the scorpion (81–130 mg kg^−1^) and tarantula samples (80–121 mg kg^−1^). Copper is involved in energy production, iron metabolism, brain development, and immune system functioning [39]. Most of the samples (crickets, locusts, worms, scorpions, ants, tarantulas, and water bugs) also contained high levels of Fe, ranging from 33 to 474 mg kg^−1^. This element is necessary for human growth and development, with it circulating oxygen throughout the body [40]. Lastly, high levels of Zn were observed for all the samples (from 100 mg kg^−1^ to 1000 mg kg^−1^). This is noteworthy, as zinc is involved in cellular metabolism (catalytic activities of enzymes, protein and DNA synthesis), immune health, and growth and development [41]. Table 8 shows that the levels of several essential elements are consistent with those previously obtained in similar samples. Differences may be attributed to different sample origin, sample preparation, etc. The systematically lower concentrations in water bugs reported previously might be a result of the legs and antennas having been removed prior to analysis, whereas the whole insect was analyzed in this work.

**Table 5:** Concentrations ± standard deviation (*n* = 2; mg kg^−1^) of essential elements in edible insects, scorpions, and tarantulas (with corresponding sample name) by ICP-MS.

Sample	Co	Cr	Cu	Fe	Mn	Mo	Se	Zn
Ants								
1	0.05241 ± 0.00074	0.12074 ± 0.00089	19.463 ± 0.042	147 ± 3	36.39 ± 0.39	0.5309 ± 0.0012	1.01 ± 0.00	113 ± 2
2	0.0519 ± 0.0032	0.114 ± 0.011	19.34 ± 0.35	146 ± 5	35.32 ± 0.41	0.5188 ± 0.0037	1.001 ± 0.012	113 ± 3
Crickets								
1	0.0615 ± 0.0053	0.0561 ± 0.0096	21.50 ± 0.60	55.80 ± 0.23	55.2 ± 1.6	0.5318 ± 0.0026	0.616 ± 0.023	191 ± 2
2	0.0611 ± 0.0017	0.0390 ± 0.0088	19.54 ± 0.90	50.5 ± 2.5	59.3 ± 1.2	0.5382 ± 0.0015	0.631 ± 0.024	197 ± 2
3	0.0557 ± 0.0012	0.0406 ± 0.0042	20.891 ± 0.048	55.2 ± 2.5	58.2 ± 1.3	0.560 ± 0.015	0.6384 ± 0.0027	202 ± 8
4	0.03363 ± 0.00092	0.0915 ± 0.0031	19.98 ± 0.33	50.9 ± 1.8	63.5 ± 1.3	0.6071 ± 0.0051	0.3087 ± 0.0035	155 ± 2
5	0.0108 ± 0.0013	0.02712 ± 0.00031	24.2 ± 1.5	54.69 ± 0.029	30.262 ± 0.052	0.723 ± 0.025	0.1234 ± 0.0064	239 ± 24
6	0.0102 ± 0.0021	0.0164 ± 0.0026	26.0 ± 2.4	49.3 ± 3.3	30.1 ± 2.9	0.755 ± 0.028	0.1311 ± 0.0017	256 ± 9
7	0.0835 ± 0.0013	0.1380 ± 0.0081	53.09 ± 0.42	83.58 ± 0.17	7.69 ± 0.20	1.4969 ± 0.0053	0.19229 ± 0.00045	150 ± 37
8	0.0880 ± 0.0070	0.122 ± 0.017	49.31 ± 0.40	68.7 ± 1.4	4.937 ± 0.048	1.531 ± 0.030	0.200 ± 0.011	135 ± 15
Locusts								
1	0.1218 ± 0.0049	0.0836 ± 0.0077	60.300 ± 0.030	90.33 ± 0.55	6.77 ± 0.18	0.678 ± 0.018	0.3341 ± 0.0071	177 ± 25
2	0.1246 ± 0.0047	0.2066 ± 0.0093	92.7 ± 7.7	164 ± 23	6.46 ± 0.18	0.574 ± 0.021	0.2160 ± 0.0068	173 ± 17
Scorpions								
1	0.683 ± 0.030	0.1658 ± 0.0023	81.8 ± 7.4	139 ± 11	166 ± 12	0.206 ± 0.014	2.136 ± 0.081	381 ± 25
2	1.0358 ± 0.0058	0.1609 ± 0.0029	130.64 ± 0.60	199 ± 4	203.0 ± 4.0	0.3230 ± 0.0040	2.62 ± 0.14	624 ± 10
3	0.4908 ± 0.0089	0.2082 ± 0.0068	128.0 ± 1.1	107 ± 1	269.1 ± 2.0	0.28131 ± 0.00035	1.512 ± 0.011	609 ± 1
4	0.1637 ± 0.0023	0.24 ± 0.16	102.87 ± 0.40	62.07 ± 0.73	68.9 ± 2.7	0.14280 ± 0.00099	1.083 ± 0.012	552 ± 2
Tarantulas								
1	0.4964 ± 0.0021	0.1479 ± 0.0029	112.33 ± 0.28	157 ± 1	735.9 ± 7.6	0.07640 ± 0.00025	1.079 ± 0.014	1011 ± 10
2	0.2461 ± 0.0042	0.3393 ± 0.0016	121.0 ± 2.8	218 ± 1	351.3 ± 1.1	0.1178 ± 0.0020	1.179 ± 0.020	821 ± 2
3	0.5862 ± 0.0049	0.2992 ± 0.0031	80.3 ± 1.2	224.2 ± 0.4	275.6 ± 4.9	0.1194 ± 0.0076	2.013 ± 0.028	734 ± 8
Water bug								
1	0.10819 ± 0.00033	0.179 ± 0.031	12.69 ± 0.36	129 ± 1	10.03 ± 0.14	0.15052 ± 0.00042	0.469 ± 0.010	147.2 ± 0.4
2	0.2217 ± 0.0054	0.211 ± 0.025	11.199 ± 0.080	474 ± 340	14.674 ± 0.053	0.2358 ± 0.0086	0.5047 ± 0.0076	119 ± 1
Worms								
1	0.04598 ± 0.00043	0.0231 ± 0.0045	16.6 ± 1.3	46.6 ± 5.0	10.72 ± 0.12	0.960 ± 0.010	0.819 ± 0.012	106 ± 6
2	0.001978 ± 0.000015	0.0203 ± 0.0019	9.12 ± 0.11	32.85 ± 0.97	19.390 ± 0.055	0.3557 ± 0.0035	0.6344 ± 0.0096	175 ± 4
3	0.048699 ± 0.000032	0.0504 ± 0.0074	9.714 ± 0.096	51.8 ± 2.4	17.7 ± 1.6	0.699 ± 0.011	0.2437 ± 0.0022	73.5 ± 3.0
4	0.01844 ± 0.00023	0.0237 ± 0.0043	19.08 ± 0.20	56.7 ± 9.7	11.762 ± 0.024	0.55998 ± 0.00063	0.10654 ± 0.00047	105 ± 1
5	0.01959 ± 0.00073	0.0798 ± 0.0096	20.02 ± 0.73	51.3 ± 1.8	11.187 ± 0.057	0.573 ± 0.014	0.1073 ± 0.0067	107 ± 3
6	0.0198 ± 0.0024	0.0223 ± 0.0067	18.45 ± 0.12	49.34 ± 0.49	11.10 ± 0.15	0.5681 ± 0.0089	0.10155 ± 0.00046	101 ± 1

**Table 6:** Concentrations ± standard deviation (*n* = 2; mg kg^−1^) of remaining elements in edible insects, scorpions, and tarantulas (with corresponding sample name) measured by ICP-MS.

Sample	Al	B	Ba	Sr
Ants				
1	99.5 ± 2.5	0.742 ± 0.024	17.54 ± 0.37	4.828 ± 0.091
2	105 ± 10	0.715 ± 0.031	14.753 ± 0.029	4.24 ± 0.23
Crickets				
1	5.49 ± 0.74	0.757 ± 0.073	0.5008 ± 0.0019	2.794 ± 0.077
2	3.06 ± 0.43	0.5543 ± 0.0056	0.464 ± 0.013	2.75 ± 0.10
3	4.3 ± 1.7	0.553 ± 0.011	0.489 ± 0.038	2.9160 ± 0.0040
4	20.4 ± 1.2	0.398 ± 0.012	0.786 ± 0.043	2.472 ± 0.048
5	12.3 ± 7.6	0.690 ± 0.043	1.06 ± 0.16	2.54 ± 0.13
6	3.79 ± 0.72	0.314 ± 0.021	0.773 ± 0.067	2.40 ± 0.33
7	6.29 ± 0.32	0.771 ± 0.018	1.4569 ± 0.0065	1.861 ± 0.039
8	4.07 ± 0.60	0.714 ± 0.041	1.171 ± 0.048	1.636 ± 0.043
Locusts				
1	55.3 ± 2.4	0.387 ± 0.031	0.9767 ± 0.0055	1.895 ± 0.028
2	119 ± 15	0.400 ± 0.020	1.298 ± 0.049	1.0888 ± 0.0059
Scorpions				
1	283 ± 17	0.659 ± 0.020	8.23 ± 0.64	4.80 ± 0.45
2	64.25 ± 0.54	0.3993 ± 0.0042	11.071 ± 0.018	10.58 ± 0.11
3	69.0 ± 2.0	0.763 ± 0.034	7.078 ± 0.062	17.99 ± 0.11
4	103.7 ± 0.5	0.429 ± 0.017	4.269 ± 0.028	5.436 ± 0.055
Tarantulas				
1	132.8 ± 0.05	0.898 ± 0.040	28.83 ± 0.17	31.22 ± 0.14
2	169 ± 3	1.0023 ± 0.0036	23.78 ± 0.41	31.08 ± 0.93
3	167 ± 1	1.068 ± 0.049	28.57 ± 0.12	21.73 ± 0.21
Water bug				
1	81.1 ± 1.4	nd	1.0085 ± 0.0063	1.2820 ± 0.0020
2	144 ± 3	nd	1.492 ± 0.016	1.424 ± 0.036
Worms				
1	14.2 ± 2.3	0.758 ± 0.012	2.238 ± 0.053	3.71 ± 0.20
2	1.406 ± 0.029	nd	0.857 ± 0.050	0.901 ± 0.016
3	14.86 ± 0.96	1.184 ± 0.028	2.22 ± 0.14	3.222 ± 0.069
4	0.490 ± 0.070	2.28 ± 0.23	3.974 ± 0.071	3.619 ± 0.064
5	1.5 ± 1.2	2.128 ± 0.070	3.83 ± 0.20	3.57 ± 0.11
6	0.4421 ± 0.0020	2.49 ± 0.36	4.003 ± 0.043	3.5216 ± 0.0024

nd: not detected.

**Table 7:** Comparison of concentrations previously reported for potentially toxic and major elements with those obtained in this work.

Insect	As (µg kg^−1^)	Cd (µg kg^−1^)	Pb (µg kg^−1^)	Ca (mg kg^−1^)	K (mg kg^−1^)	Mg (mg kg^−1^)	Na (mg kg^−1^)	Reference
Cricket (*n* = 3)	300–400	22–28	n.d.-19	612–2630	8100–13,500	740–1880	1130–15,900	[27]
Cricket (*n* = 8)	3.6–21.1	13.8–76.6	16.7–96.9	411–1798	5263–9116	727–1316	1162–2874	This work
Locust (*n* = 1)	300	n.d.	16	864	6060	490	2160	[27]
Locust (*n* = 2)	35.0–47.4	31.8–40.9	50.2–73.4	481–559	6350–6510	658–749	860–937	This work
Mealworm (*n* = 3)	200–620	30–200	n.d.-100,000	941–2380	7560–10,000	870–1450	1290–1510	[27]
Mealworm (*n* = 5)	25.7–39.7	79.3–83.8	6.0–10.8	401–788	4474–8533	1343–3074	521–982	This work
Silkworm (*n* = 1)	200	n.d.	9	622	1090	480	3650	[27]
Silkworm (*n* = 1)	470	53.9	47.3	1132	7462	2565	126	This work
Water bug (*n* = 5)				320.9–561.5	220–346	336–420	197.4–286.2	[23]
Water bug (*n* = 2)				632–855	3950–4041	614–695	3150–5113	This work

**Table 8:** Comparison of concentrations (mg kg^−1^) previously reported for essential elements with those obtained in this work.

Insect	Co	Cr	Cu	Fe	Mn	Mo	Se	Zn	Reference
Cricket (*n* = 3)	n.d.-0.045	0.174–0.267	16.3–23.9	40.9–80.7	9.62–45.8	0.327–17.2	0.474–0.899	125–173	[27]
Cricket (*n* = 8)	0.0102–0.0880	0.0164–0.1380	19.54–53.09	49.3–83.6	7.7–63.5	0.560–1.531	0.123–0.638	135–256	This work
Locust (*n* = 1)	n.d.	0.140	35.7	42.4	4.44	0.734	0.307	140	[27]
Locust (*n* = 2)	0.1218–0.1246	0.0836–0.2066	60.3–92.7	90.3–164	6.46–6.77	0.574–0.678	0.216–0.334	173–177	This work
Mealworm (*n* = 3)	0.018–0.033	0.164–0.624	11.3–28.2	36.2–253	5.36–410	0.37–0.897	0.474–0.547	93.8–126	[27]
Mealworm (*n* = 5)	0.01844–0.04870	0.0223–0.0798	9.71–20.02	46.6–56.7	10.7–17.7	0.560–0.960	0.102–0.819	73.5–107	This work
Silkworm (*n* = 1)	n.d.	0.054	3.90	11.7	5.71	0.118	0.357	38.6	[27]
Silkworm (*n* = 1)	0.001978	0.0203	9.12	32.85	19.390	0.3557	0.6344	175	This work
Water bug (*n* = 5)			22.2–420	253–1121	19.8–42.2			49.8–72.2	[23]
Water bug (*n* = 2)			11.2–12.7	129–474	10.03–14.67			119–147	This work

## 4. Conclusions

This study addresses the assessment of potentially toxic, major, and essential elements (19 trace elements in total) in a variety of edible insects, scorpions, and tarantulas by ICP-MS. The method accuracy was verified by analyzing several insect certified reference materials. Based on the provisional tolerable daily intake (PTDI) values for As, Cd, and Pb, it was concluded that most of the samples were safe to consume for both children and adults at relatively high amounts (number of individuals ranging from 1000 to 100,000 insects per day). However, the maximum number of scorpions that can be safely consumed is much lower (up to ~30 for adults and up to 10 for children), while in the case of tarantulas, consumption of only 1 individual per day is recommended for children and 2–3 for adults.

Lastly, high levels of Cu were found in the tarantula and scorpion samples as well as K, Na, Fe, and Zn in most of the insect samples, suggesting that edible insects have a nutritional value. 

A limitation of this study is that it included no information available on the insects’ developmental stage, environmental exposition, bioaccumulation of pesticides and chemicals, which parts of the insects were used in previous studies, and the origin of the following samples: crickets 5–6, locusts 1–2, and water bug 1–2. 

The work will be pursued by increasing the replicate size in order to assess the samples’ heterogeneity and also extending the study to other edible insect species in order to provide a more extensive database regarding the benefit–risk balance related to trace elements via consumption of this type of foodstuffs.

## Figures and Tables

**Table 1:** Operating conditions of the ICP-MS/MS instrument (Agilent 8900).

Parameter	Setting
RF power	1550 W
Plasma gas flow rate (Ar)	15.0 L min^−1^
Auxiliary gas flow rate (Ar)	0.9 L min^−1^
Nebulizer gas flow rate (Ar)	~1.0 L min^−1^
Collision gas flow rate (He)	~5.0 mL min^−1^
Sampling/skimmer cones	Nickel
Monitored isotopes	No gas mode: ^11^B, ^75^As, ^111^Cd, ^137^Ba, ^208^Pb
	Collision mode (He): ^23^Na, ^24^Mg, ^27^Al, ^39^K, ^44^Ca, ^52^Cr, ^55^Mn, ^56^Fe, ^59^Co, ^63^Cu, ^66^Zn, ^77^Se, ^88^Sr, ^95^Mo
	Internal standards: ^89^Y, ^185^Re, ^209^Bi

**Table 2:** List of edible insects, scorpions, and tarantulas samples used in the study with corresponding code number, sample name, ingredients, and origin (if available).

Sample Name	Insect	Species	Information	Origin
Ants 1	Columbia ants	*Atta laevigata*	With salt	Columbia
Ants 2	Columbia ants	*Atta laevigata*	With salt	Columbia
Crickets 1	Crickets	*Acheta domesticus*	Whole dehydrated crickets	Toulouse, France
Crickets 2	Crickets	*Acheta domesticus*	Whole dehydrated crickets	Toulouse, France
Crickets 3	Crickets	*Acheta domesticus*	Whole dehydrated crickets	Toulouse, France
Crickets 4	Crickets	*Gryllus bimaculatus*	-	Thailand
Crickets 5	Crickets	N/A	Whole dehydrated crickets	N/A
Crickets 6	Crickets	N/A	Whole dehydrated crickets	N/A
Crickets 7	Crickets	N/A	Whole dehydrated crickets	Netherlands
Crickets 8	Crickets	N/A	Whole dehydrated crickets	Netherlands
Locusts 1	Locusts	*Locusta migratoria*	With salt	N/A
Locusts 2	Locusts	*Locusta migratoria*	With salt	N/A
Scorpions 1	Black scorpions	*Heterometrus longimanus*	With salt	Thailand
Scorpions 2	Black Asian forest scorpions	*Heterometrus longimanus*	With salt	Thailand
Scorpions 3	Black Asian forest scorpions	*Heterometrus longimanus*	With salt	Thailand
Scorpions 4	Black Asian forest scorpions	*Heterometrus longimanus*	With salt	Thailand
Tarantulas 1	Tarantula	*Haplopeima albostriatum*	-	Thailand
Tarantulas 2	Tarantula	*Haplopeima albostriatum*	-	Thailand
Tarantulas 3	Tarantula	*Haplopeima albostriatum*	-	Thailand
Water bug 1	Giant water bug	*Nepidae*	With oil and salt	N/A
Water bug 2	Giant water bug	*Nepidae*	With oil and salt	N/A
Worms 1	Mealworms	*Tenebrio molitor*	-	EU
Worms 2	Silk worms	*Bombyx mori*	-	Thailand
Worms 3	Morio worms	*Zophobas morios*	With salt	Thailand
Worms 4	Mealworms	*Tenebrio molitor*	Whole dehydrated mealworm	Toulouse, France
Worms 5	Mealworms	*Tenebrio molitor*	Whole dehydrated mealworm	Toulouse, France
Worms 6	Mealworms	*Tenebrio molitor*	Whole dehydrated mealworm	Toulouse, France

## Data Availability

The original contributions presented in the study are included in the article/Supplementary Materials; further inquiries can be directed to the corresponding author.

## Supplementary Materials

The following supporting information can be downloaded at: https://www.mdpi.com/article/10.3390/foods13152353/s1, Table S1: Statistical comparison of measured concentrations in insect CRMs with their certified values.
