# Supplementary material for: Multi-Elemental Analysis of Edible Insects, Scorpions, and Tarantulas from French (Online) Market and Human Health Risk Assessment Due to Their Consumption: A Pilot Study
Source: Foods. 2024 Jul 26;13(15):2353. doi: 10.3390/foods13152353 (PMC11311618; doi:10.3390/foods13152353)
Supplement: Supplementary file 1 [file foods-13-02353-s001.zip › foods-3090509-supplementary.pdf]

Supplementary Material for

Multi-Elemental Analysis of Edible Insects, Scorpions, and Tarantulas from French (Online) Market and Human Health Risk Assessment Due to Their Consumption: A Pilot Study

Yulianna Holowaty [1], Axelle Leufroy [2], Clément Mazurais [2], Diane Beauchemin [1] and Petru Jitaru [2,*]

[1] Department of Chemistry, Queen's University, 90 Bader Lane, Kingston, ON K7L 3N6, Canada; 18yzh@queensu.ca (Y.H.); diane.beauchemin@queensu.ca (D.B.)
[2] Laboratory for Food Safety, University Paris East Creteil, Anses, F-94700 Maisons-Alfort, France; axelle.leufroy@anses.fr (A.L.); clement.mazurais@anses.fr (C.M.)
* Correspondence: petru.jitaru@anses.fr

Table S1. Statistical comparison of measured concentrations in insect CRMs with their certified values.

CRM	Al		As		B		Ba		Ca	
	Δ_m	U_Δ	Δ_m	U_Δ	Δ_m	U_Δ	Δ_m	U_Δ	Δ_m	U_Δ
**BFLY-1**	16	40	0.027	0.040	0.92	2.4	0.82	2.3	150	920
**KRIK-1**	1.3	3.7	0.0056	0.037	0.050	1.3	0.029	0.047	49	180
**VORM-1**	4.3	7.6	0.0042	0.021	0.73	1.5	0.018	0.98	10	88
**CRM**	**Cd**		**Co**		**Cr**		**Cu**		**Fe**	
	Δ_m	U_Δ	Δ_m	U_Δ	Δ_m	U_Δ	Δ_m	U_Δ	Δ_m	U_Δ
**BFLY-1**	0.0064	0.061	0.042	0.029	0.36	0.39	0.37	2.5	5.5	49
**KRIK-1**	0.0073	0.014	0.0030	0.018	0.024	0.040	0.062	4.9	4.4	7.6
**VORM-1**	0.0031	0.022	0.0036	0.030	0.0027	0.030	0.27	2.7	4.3	21
**CRM**	**K**		**Mg**		**Mn**		**Mo**		**Na**	
	Δ_m	U_Δ	Δ_m	U_Δ	Δ_m	U_Δ	Δ_m	U_Δ	Δ_m	U_Δ
**BFLY-1**	1300	970	670	690	8.2	23	0.14	0.21	332	333
**KRIK-1**	480	640	120	280	0.048	1.4	0.015	0.17	99	420
**VORM-1**	560	640	35	570	0.35	2.2	0.039	0.39	92	340
**CRM**	**Pb**		**Se**		**Sr**		**Zn**			
	Δ_m	U_Δ	Δ_m	U_Δ	Δ_m	U_Δ	Δ_m	U_Δ		
**BFLY-1**	0.011	0.078	0.086	0.085	0.98	5.1	8.2	8.9		
**KRIK-1**	0.0037	0.0084	0.033	0.13	0.016	0.28	1.8	16		
**VORM-1**	0.00096	0.019	0.0016	0.037	0.062	1.1	4.6	16		
